# Examining health disparities and characteristics in general practice utilization: based on outpatient data from 2014 - 2018 in Shanghai

**DOI:** 10.1186/s12875-020-01146-5

**Published:** 2020-04-29

**Authors:** Jianwei Shi, Chunhua Chi, Xin Gong, Chen Chen, Wenya Yu, Jiaoling Huang, Liang Zhou, Ning Chen, Yan Yang, Qian Liu, Zhaoxin Wang

**Affiliations:** 1 School of Public Health, Shanghai Jiaotong University School of Medicine, 227 South Chongqing Rd, Shanghai, 200025 China; 2 Yangpu Hospital, Tongji University School of Medicine, Shanghai, 200090 China; 3 General Practice Department, Peking University First Hospital, Beijing, 100034 China; 4 School of Medicine, Tongji University, Shanghai, 200092 China; 5 Pengpuxincun Community Health Service Center, Jingan District, Shanghai, 200080 China; 6 School of Economics & Management, Tongji University, Shanghai, 200092 China; 7 General Practice Center, Nanhai Hospital, Southern Medical University, Foshan, 528244 China

**Keywords:** Community health, Disease, Evidence-based

## Abstract

**Background:**

Since 2000, China has been developing primary care institutions to serve as the gateway to the healthcare system. However, the investment of resources in primary care institutions is not based on the actual medical demands of the public. This study analysed primary care utilization to provide targeted guidance for the improvement of primary healthcare delivery in China.

**Methods:**

We extracted outpatient visit data from all community healthcare centres in Shanghai from 2014 to 2018. Diseases were then classified according to ICD-10 codes. The disease spectrum (frequency, proportion, rank) was stratified by sex, age, and region.

**Results:**

Most primary care outpatients were female (58.20%), 60–79 years old (57.91%), and in suburban regions (62.18%). Chronic diseases accounted for the majority (91.41%). Hypertension, chronic ischaemic heart disease, diabetes, and acute upper respiratory tract infections were the top four disorders for primary care visits regardless of sex. In the group aged 0–18 years, symptoms, signs and abnormal clinical and laboratory findings not elsewhere classified accounted for 37.96% of the top 20 reasons. Acute upper respiratory tract infections were the most common diseases in the groups aged 0–18 (11.20%) and 19–39 (11.14%) years. However, hypertension was the most common disease in the group aged > 39 years old (> 20%). There were more outpatients with respiratory and digestive diseases in suburban areas than in urban areas. In addition, problems associated with medical equipment and other healthcare deficiencies were relatively more common in suburban areas (suburban: 4.13%, rank 5; urban: 2.29%, rank 10).

**Conclusions:**

To meet the patients’ needs and to develop the primary care system, the Shanghai government should focus on diseases with regionally high proportions. Disease diagnosis and treatment should be improved in the younger and suburban populations.

## Background

In China, there is no strict referral mechanism for Chinese residents seeking medical treatment [1]. The public tends to go directly to secondary and tertiary public hospitals instead of primary care institutions because of the high quality care in hospitals. To promote the medical capabilities of community healthcare institutions, China is actively learning from Western countries, enacting healthcare reform and vigorously developing general practices in communities. In the community healthcare institutions in China, usually the general practitioners provided health service for the patients, and the patients should pay for the service each time when they seek the treatment. For patients with various health insurance, namely urban social insurance for workers, urban social insurance for citizens, new rural cooperative medical insurance, self-paying or other, the reimbursement ratio showed a decreasing tendency in that order. However, the current health insurance hasn’t played important role in restricting patients’ behaviour [1, 2]. The ageing of society and the accompanying high incidence rates of chronic diseases [3] have led primary healthcare institutions to prioritize the prevention and treatment of non-communicable diseases (NCDs). This finding is consistent with the results of the Global Burden of Disease Study 2013 [4], which showed that the disease burden was dominated by NCDs, affecting years lived with disabilities (YLDs) in all regions except sub-Saharan Africa. In the past 20 years, NCDs have been the focus of researchers and policy makers worldwide.

However, at present, as it develops a national primary healthcare system, the Chinese government usually provides financial support for the prevention and treatment of certain diseases with high incidence rates in Western countries, such as hypertension and diabetes [1, 2]. However, there is no accurate evidence supporting this prioritization of conditions and the consequent allocation of medical resources in community healthcare institutions. The limitations of the existing studies are as follows: first, there is a lack of high-quality institution-level disease spectrum evidence [3, 5]. Usually, the conclusions drawn were primarily based on data from the CDC surveillance system in China [1–3]. However, these data are sampled and are usually targeted at the cause of death and the morbidity rates of specific diseases, including some infectious diseases and a small proportion of chronic diseases, such as cancer. Second, although existing research has studied disease patterns, few have explored the characteristics of those patients who primarily seek service in community institutions [5–8]. To increase the accuracy of the morbidity rates of broader categories of infectious diseases and NCDs, increasing numbers of studies are beginning to focus on the high-level data, such as inpatient and outpatient electronic health records (EHRs), which are collected from healthcare institutions. Yu et al. elucidated the spectrum and characteristics of severe NCDs in eastern coastal China by analysing the longitudinal EHRs from inpatients in 12 general tertiary hospitals [5]. In 2017, the Health Bureau of Chongqing city in China analysed the characteristics of inpatients with cancer based on EHRs from all secondary and tertiary hospitals [9]. However, there is a lack of service demand and healthcare delivery in primary care institutions.

To the best of our knowledge, our study is the first analysis of the distribution of all categories of diseases by analysing the vast quantity of outpatient data from all community health care institutions in Shanghai. As the fastest growing region in China’s economy, Shanghai places great emphasis on general practice and public health, and community health service development is also of paramount importance. The characteristics of this city are as follows: first, comparatively, the overall health of its residents are similar to that of individuals in developed countries; second, the diversity within groups, such as urban vs. rural inhabitants and individuals of different sexes and ages, may contribute to differences in the utilization of primary care institutions [10]. Therefore, it is very important to analyse primary care utilization to inform the allocation of resources and support the development of functional community healthcare centres in this region.

The aim of this study was to explore the distribution characteristics of all diseases treated in community health care centres in Shanghai in the past 5 years, including the proportions of acute diseases, chronic diseases, injuries, poisonings, pregnancies and childbirths, and perinatal diseases, in different sexes, age groups and regions. The results of this study may provide guidance regarding the allocation of medical resources for the treatment, prevention and control of key diseases for Shanghai and other developed regions. This information may be useful for enriching regional and national policy debates to improve primary healthcare.

## Methods

### Data source

In China, Shanghai is one of the most developed areas in terms of information technology and medical care. However, the early medical data are relatively incomplete, and the collection and storage of data were not perfect. Due to the slow initiation and development of primary care institutions, the related information system was the last to be constructed among the medical information databases in Shanghai. After 2010, the Shanghai municipal government gradually came to appreciate the importance of establishing this medical database. During 2014–2018, there were averagely around 240 community healthcare centres in shanghai, with over 40,000,000 outpatient visits and over 100 million pieces of information generated and stored annually. By 2012, the outpatient information system platform for community healthcare institutions had been fundamentally improved. Therefore, to ensure data quality, the data from 2014 to 2018 from this database were included in this study.

The outpatient system platform for community healthcare institutions in Shanghai has the following characteristics: (1) it includes the demographics and medical information of all patients in all community health care centres in Shanghai and (2) diagnoses are recorded using the ICD-10 codes. The outpatient visit data are first collected by each community healthcare institution and then reported to the regional health information department. Finally, they are collected by the health information department of the Shanghai Health Committee, which manages the community healthcare outpatient database in Shanghai.

### Study population

In this study, to ensure the accuracy and consistency of the data, all data pertaining to patients who visited the community healthcare centres in Shanghai were included from January 1, 2014, to December 31, 2018. Demographic datasets were composed of patient sex, age, and region. The ICD-10 diagnosis code for each outpatient visit was also included.

Data in this study were from the outpatient population (*n* = 301,651,169) in all community health service centres (number of community health care centres, n (2014) = 221, n (2015) = 268, n (2016) = 266, n (2017) = 271, n (2018) = 284) in Shanghai. Incomplete and erroneous data were removed, and data from 283,566,225 visits were finally included. Medical information was extracted from the outpatient medical records, and demographic characteristics were stratified by sex and age to construct the outpatient population dataset. Finally, the patients were grouped according to the body systems affected to generate the disease spectrum dataset.

### Statistical analysis

This study analysed the spectrum of all outpatient visits in all community health service centres in Shanghai, including the frequency, proportion and rank of the affected system stratified by patient sex (male and female), age (0–18 years old, 19–39 years old, 40–59 years old, 60–79 years old, and older than 80 years) and region (urban and suburban areas). Furthermore, we identified the top 20 most common disorders in each group in this study.

## Results

### Description of outpatients from all community health care centres between 2014 and 2018 in Shanghai

The demographics of all the extracted outpatients from community healthcare centres are presented in Table 1. The number of visits made by female outpatients was greater than that made by male outpatients (female: *n* = 165,030,342, 58.20%; male: *n* = 118,535,883, 41.80%), and both sexes showed an increasing tendency to visit the community healthcare centres from 2014 to 2018. Regarding age, the majority of the outpatients were 60–79 years old (57.91%), and except among the group older than 80 years, the number of outpatient visits slightly increased in each age group from 2014 to 2018. The majority of the outpatient visits were in the suburban region (62.18%), and the number of outpatient visits in both the urban and suburban regions increased from 2014 to 2018.

**Table 1:** Description of outpatients in all community health care centres between 2014 and 2018 in Shanghai

Classification	2014		2015		2016		2017		2018		Total	
	N	%	N	%	N	%	N	%	N	%	N	%
**Sex**												
Male	16,605,934	40.50	22,034,737	41.42	24,301,296	41.59	27,104,224	42.20	28,489,692	42.71	118,535,883	41.80
Female	24,395,000	59.50	31,168,776	58.58	34,130,845	58.41	37,125,869	57.80	38,209,852	57.29	165,030,342	58.20
**Age (years)**	41,000,934		53,203,513		58,432,141		64,230,093		66,699,544		283,566,225	
0–18	339,857	0.83	436,081	0.82	506,072	0.87	705,225	1.10	832,174	1.25	2,819,409	0.99
19–39	997,509	2.43	1,568,214	2.95	1,856,866	3.18	2,106,804	3.28	2,275,357	3.41	8,804,750	3.11
40–59	4,878,757	11.9	7,138,358	13.42	8,235,854	14.09	9,465,784	14.74	10,383,416	15.57	40,102,169	14.14
60–79	23,126,315	56.4	30,213,325	56.79	33,763,085	57.78	37,535,061	58.44	39,580,498	59.34	164,218,284	57.91
Above 80	11,658,496	28.43	13,847,535	26.03	14,070,264	24.08	14,417,219	22.45	13,628,099	20.43	67,621,613	23.85
**Region**												
Urban	17,501,250	42.69	19,190,524	36.07	21,024,441	35.98	23,608,055	36.76	25,919,069	38.86	107,243,339	37.82
Suburban	23,499,684	57.31	34,012,989	63.93	37,407,700	64.02	40,622,038	63.24	40,780,475	61.14	176,322,886	62.18
**Total**	41,000,934	100.00	53,203,513	100.00	58,432,141	100.00	64,230,093	100.00	66,699,544	100.00	283,566,225	100.00

### Distribution of disease systems for outpatient visits stratified by sex in all community health care centres in Shanghai between 2014 and 2018

**Fig. 1:**
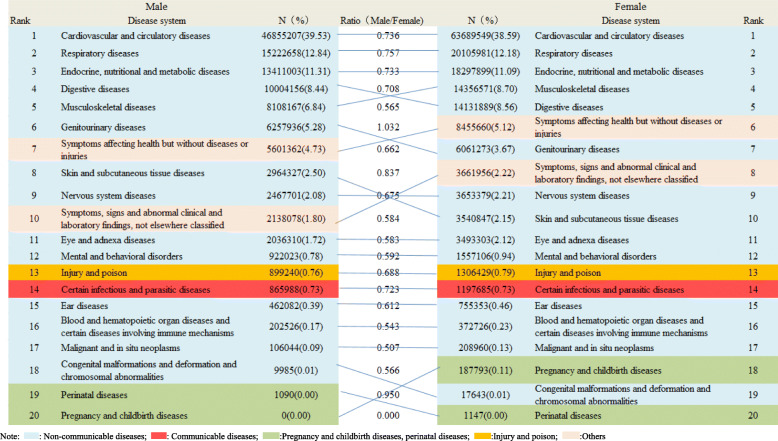
Distribution of disease system for outpatient visits stratified by sex in all community health care centres in Shanghai between 2014 and 2018

As shown in Fig. 1, the overall distribution of various diseases system rank for outpatient visits was similar in men and women. However, except for genitourinary diseases (male vs. female: 5.28% vs. 3.67%), female outpatients predominated in all groups (ratio: male vs. female: 0.000–1.032). Cardiovascular and circulatory diseases (male: *n* = 46,855,207, 39.53%; female: *n* = 63,689,549, 38.59%), chronic respiratory diseases (male: *n* = 15,222,658, 12.84%; female: *n* = 20,105,981, 12.18%), endocrine and nutritional metabolic diseases (male: *n* = 13,411,003, 11.31%; female: 18,297,899, 11.09%), digestive diseases (male: *n* = 10,004,156, 8.44%; female: 14,131,889, 8.56%), and musculoskeletal diseases (male: *n* = 8,108,167, 6.84%; female: 14,356,571, 8.70%) were the most common disorders for outpatient visits.

**Fig. 2:**
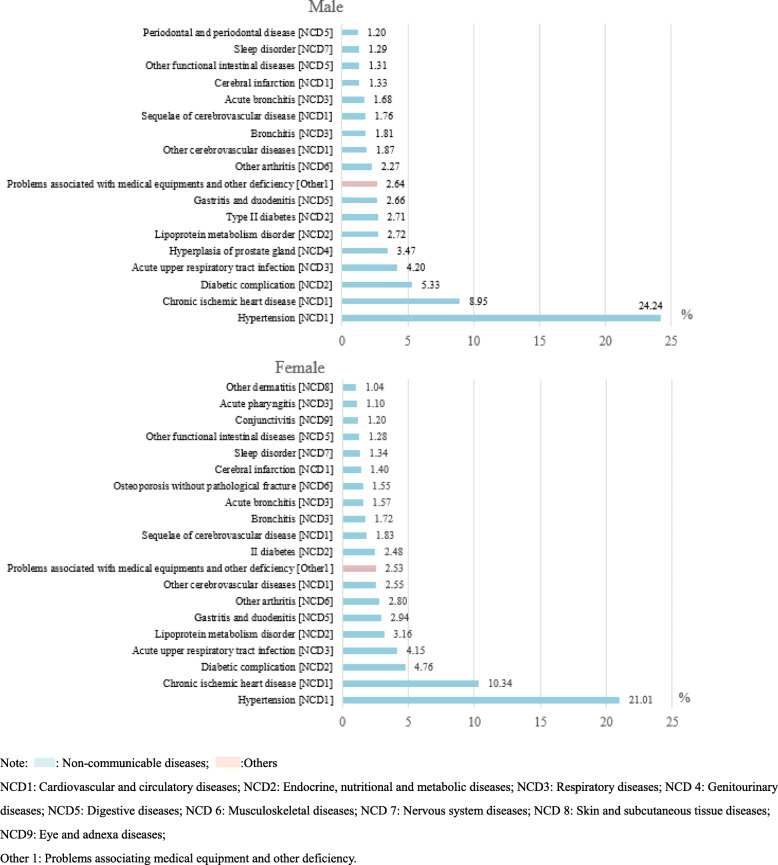
Top 20 most common disorders for outpatient visits stratified by sex in all community health care centres between 2014 and 2018 in Shanghai

Furthermore, we analysed the commonly occurring disorders for outpatient visits stratified by sex (Fig. 2). Hypertension, chronic ischaemic heart disease, diabetic complications, and acute upper respiratory tract infection were the top four diseases listed in descending order for both men and women. However, the proportions of hypertension (male VS female: 24.24% VS 21.01%) and diabetes (male VS female: 2.71% VS 2.48%) outpatients visits were higher among males than among females. In addition, gingivitis and periodontal diseases were among the top 20 disorders for outpatient visits among men (*n* = 1,426,288, 1.20%) but not among women. However, functional intestinal disorders (*n* = 2,120,092, 1.28%) and conjunctivitis (n = 1,975,173, 1.20%) were unique to women. Meanwhile, the ranks of lipoprotein metabolic disorder, gastritis and duodenitis and arthritis were slightly higher among women than among men.

### Distribution of disease systems for outpatient visits stratified by age in all community health care centres in Shanghai between 2014 and 2018

As shown in Table 2, symptoms affecting their health but without diseases or injuries were the most and second most common reasons for visits among the groups aged 0–18 years and 19–39 years, respectively (0–18: n = 1,090,178, 38.67%; 19–39: n = 1,206,583, 13.70%). However, unlike in the younger age groups, in those older than 40 years, cardiovascular and circulatory diseases and endocrine and nutritional metabolic diseases were the most common. For instance, cardiovascular and circulatory diseases were the most common disease system for visits and accounted for 33.33, 40.61, 43.86% of the visits in the groups aged 40–59 years, 60–79 years, and older than 80 years, respectively. Visits for chronic respiratory diseases (ranked 1 to 2, accounting for 33.09 to 10.48% of all outpatient visits as age increased) and digestive diseases (ranked 3 to 5, accounting for 12.96 to 7.51% of all outpatient visits as age increased) were both common in all age groups. Furthermore, compared to the proportion in the group of outpatients aged 0–18 years, the proportions of musculoskeletal diseases were relatively higher in the other age groups, ranking fourth to fifth. Among outpatients of all ages, the proportion of those with neoplasms in the community was relatively low. Comparatively speaking, the proportion of outpatient visits for injuries and poisoning and the proportion for communicable disorders were higher in the group of outpatients aged 0–18 years.

**Table 2:**
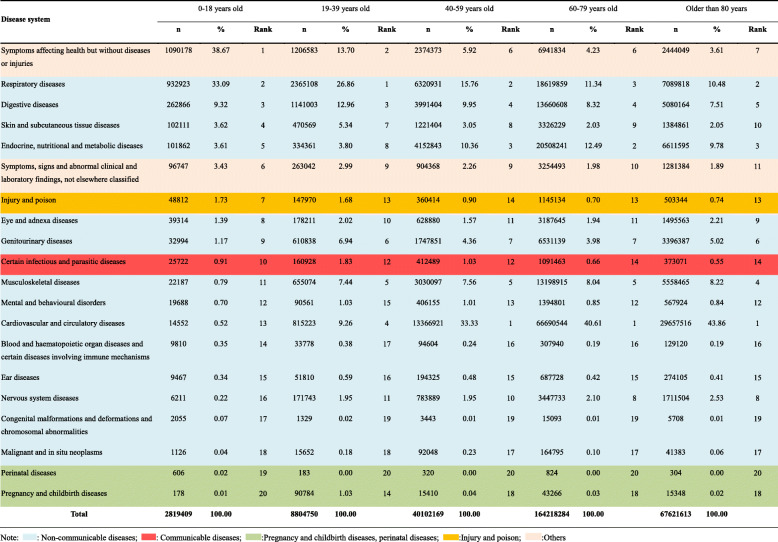
Distribution of disease system for outpatient visits to all community health care centres in Shanghai between 2014 and 2018 stratified by age group

Note: : Non-communicable diseases; : Communicable diseases; :Pregnancy and childbirth diseases, perinatal diseases; :Injury and poison; :Others

**Fig. 3:**
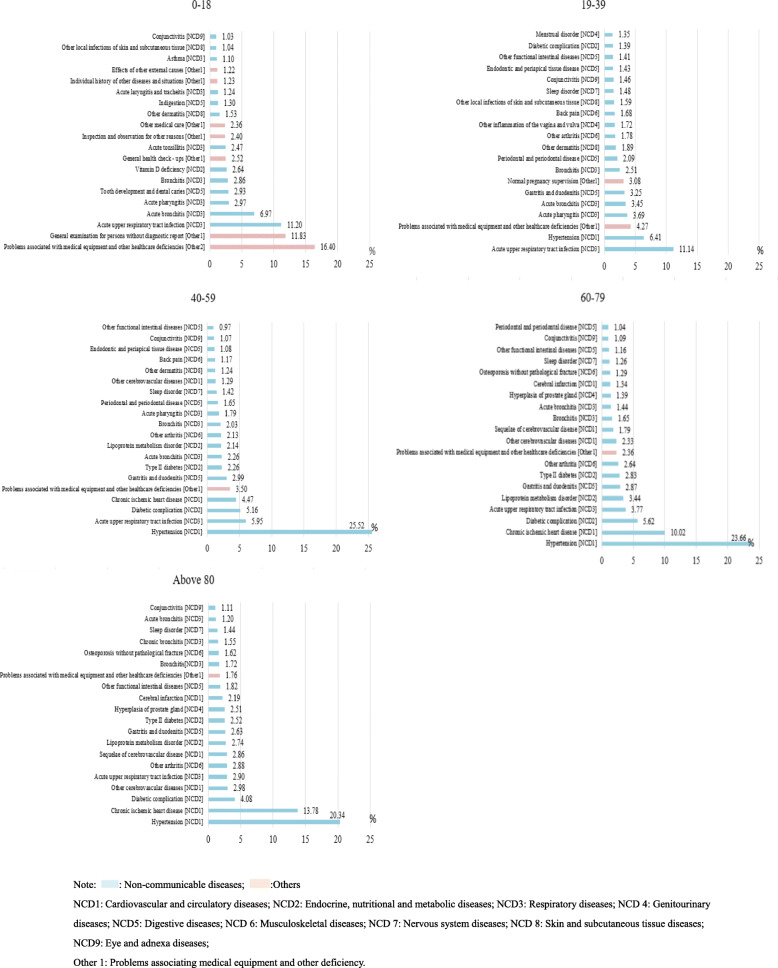
Top 20 disorders for outpatient visits stratified by age in all community health care centres between 2014 and 2018 in Shanghai

Figure 3 shows that in the group of outpatients aged 0–18 years, symptoms that affected health without diseases or injuries accounted for 37.96% of the top 20 disorders for outpatient visits. Among NCDs, acute upper respiratory tract infections were most common among the groups of outpatients aged 0–18 (*n* = 315,876, 11.20%) and 19–39 years (*n* = 980,678, 11.14%). However, as age increased, the number of outpatients with this condition decreased. Normal pregnancy supervision (*n* = 271,510, 3.08%, rank 7), inflammation of the vagina and vulva (*n* = 151,058, 1.72%, rank 12), and menstrual disorders (*n* = 118,727, 1.35%, rank 20) were more common in the group aged 19–39 years than in the other groups. Among the patients aged > 39 years, hypertension was the most common disease (accounting for 20%). Meanwhile, diabetes and chronic ischaemic heart disease were also more relatively common in that age group. In addition, gastritis and duodenitis were relatively more common in the age groups older than 18 years (ranked 6 to 9).

### Distribution of disease systems for outpatient visits stratified by region in all community health care centres in Shanghai between 2014 and 2018

As shown in Table 3, all disease system for outpatient visits were more common in the suburban regions than in the urban areas except for injuries and poisoning (ratio: urban/suburban: 2.122), pregnancy and delivery-related disorders (ratio: urban/suburban: 1.318), and congenital malformations and deformations and chromosomal abnormalities (ratio: urban/suburban: 1.357). However, endocrine and nutritional metabolic diseases (suburban vs urban: 5.84% vs 3.50%); other diseases (disorders affecting health but without diseases or injuries) (suburban vs urban: 13.42% vs 12.65%); skin and subcutaneous diseases (suburban vs urban: 2.52% vs 1.93%); symptoms, signs, and clinical and laboratory abnormalities not classified elsewhere (suburban vs urban: 2.23% vs 1.73%); mental and behavioural disorders (suburban vs urban: 0.93% vs 0.78%), and certain infectious and parasitic diseases (suburban vs urban: 0.77% vs 0.66%) were more common in suburban areas than in urban areas.

**Table 3:**
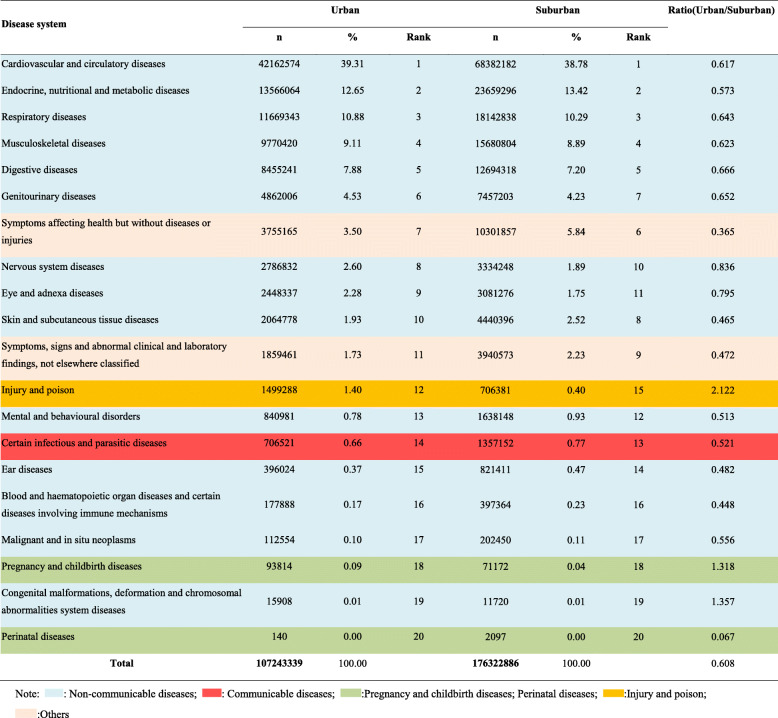
Distribution of disease systems for outpatient visits to all community health care centres in Shanghai between 2014 and 2018 stratified by region

Note: : Non-communicable diseases; : Communicable diseases; :Pregnancy and childbirth diseases; Perinatal diseases; :Injury and poison; :Others

**Fig. 4:**
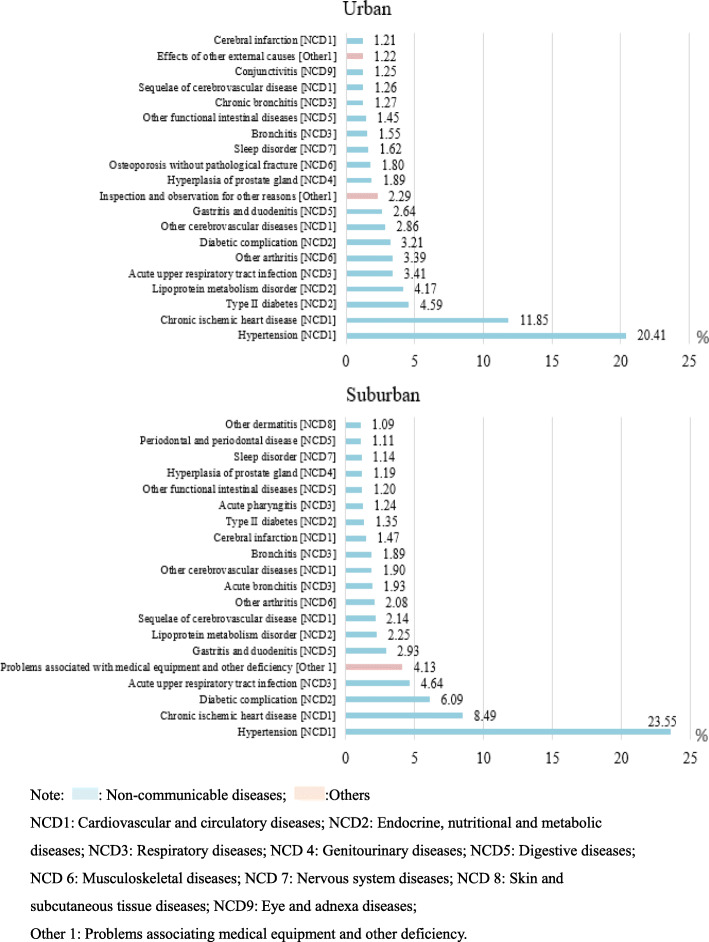
Top 20 disorders for outpatient visits stratified by region in all community health care centres between 2014 and 2018 in Shanghai .

As shown in Fig. 4, hypertension and chronic ischaemic heart disease ranked 1 and 2 in both urban and suburban areas. Diabetes (*n* = 4,922,493, 4.59%) and lipoprotein metabolism disorders (n = 4,472,250, 4.17%) were more common in urban areas than in suburban areas. Hyperplasia of the prostate gland was also more common in urban areas than in suburban areas (*n* = 2,022,831, 1.89%). However, acute upper respiratory tract infections and gastritis and duodenitis were more common in suburban areas than in urban areas. For instance, gastritis and duodenitis ranked 9th (n = 2,830,336, 2.64%) in urban areas but 6th (*n* = 5,173,555, 2.93%) in suburban areas. In addition, problems associated with medical equipment and other healthcare deficiencies were more common in suburban areas than in urban areas (suburban: 4.13%, rank 5; urban: 2.29%, rank 10).

## Discussion

By extracting disease patterns and characteristics from the outpatient visit records of community healthcare centres, this study provides evidence that can be used to guide decisions about the allocation of healthcare resources, enabling the delivery of services by community healthcare institutions and more successfully shaping them as the gatekeepers of the healthcare system. In our study, the population of Shanghai was relatively stable from 2014 to 2018. However, the results showed that the number of outpatient visits substantially increased. In addition, we found that the proportion of outpatients over 80 years decreased as time passed, but proportion of younger patients increased, indicating that a diverse population was utilizing these institutions. In addition, the proportion of visits in increased in suburban areas but decreased in urban areas. These may be explained by the improvements made to the referral system in Shanghai.

Overall, visits for NCDs accounted for the majority of visits in our study. The fact that the proportions of visits for cardiovascular and circulatory diseases and chronic respiratory diseases were the highest may be associated with air pollution in Shanghai, as it has been proposed by many researchers that exposure to high levels of ambient particulate matter (PM) is related to the incidence of these diseases in China [11, 12]. Among cardiovascular diseases, hypertension and chronic ischaemic heart disease were the most common reasons outpatients sought care in community healthcare centres, especially among outpatients older than 18 years. This is consistent with the morbidity rates of chronic diseases in the “Monitoring report of chronic diseases and their risk factors in Shanghai” released by the Shanghai CDC, which reported that 28.4% of residents over 18 years old in Shanghai had hypertension in 2012 [13, 14]. These diseases should be paid particular attention when improving the medical capacity of community healthcare centres. Chronic respiratory diseases were common in both males and females and in various age groups (ranked 2). In the group aged < 39 years, the situation was more serious. In other cities in China, chronic respiratory diseases are severe and common due to air pollution [15–17], and they have constituted a serious problem for many years [18, 19]. Data from a systematic review study with source from 17 provinces showed that for the outpatients between 0 and 18 years in community healthcare institutions, the number of respiratory diseases outpatients were the top one [20]. However, it ranked 2 for this age group in our study. This difference can be explained that many of the community health centres in Shanghai didn’t have pediatric departments, and patients would love to choose the higher level hospitals due to its good access to these medical service. By comparing with other counties, we found that in the GBD study in 327 regions, the global incidence and prevalence rates of respiratory diseases, including chronic respiratory diseases and acute upper respiratory infections, were not the highest among all studied conditions. Additionally, in high-income developed countries, the estimated loss of YLDs due to respiratory diseases was much lower than that in low-income developed countries, regardless of sex or age [21]. This may be due to air pollution, which is positively associated with the admission of respiratory patients in developing countries among different age groups; this association is especially strong in vulnerable groups (children and elderly individuals) [22–24]. However, the fact that community health centre visits were frequently for acute upper respiratory tract infections was ignored in terms of public healthcare resource allocation [25]. In contrast, chronic obstructive pulmonary disease and community-acquired pneumonia, which were rarely the reasons for visits to community healthcare centres, were afforded substantial attention from the government. This indicates that more resources should be allocated to the treatment of acute respiratory infections, especially in the relatively younger population. In this study, diabetes was more common in the group aged over 40 years than in the younger age groups. This was in accordance with the healthcare resource allocation policy. The group aged 0–30 years was more likely to suffer from digestive diseases than other age groups. The high proportion of outpatient visits for digestive diseases may be due in large part to the irregular eating habits that form part of the fast-paced lifestyle in Shanghai [26, 27]. However, digestive diseases have not received much attention in public health policies.

In recent years, NCDs have become the priority of the government [28, 29]. Communicable, maternal, and neonatal disease only accounted for a small proportion of the total visits from 2014 to 2018 in Shanghai. Comparatively, the global incidence of communicable diseases was much higher according to the Global Burden of Disease Study 2017 based on 354 regions [30] than that in Shanghai in our study. Major success in preventing and treating these diseases in Shanghai may be primarily attributed to the efforts to prevent and control HIV, childhood infectious diseases, pneumonia, etc. The treatment and prevention of these diseases were the major objectives of the “Public Health Action of the Shanghai Government” (launched by the government to prevent major public health problems following the SARS outbreak in 2003 and focused on the control of both communicable diseases and NCDs) [29]. Moreover, in 2017, efforts to prevent and control communicable diseases continued in Shanghai, with the release of the “Regulation on the Prevention and Control of Infectious Diseases in Shanghai” [31]. However, we cannot deny that communicable diseases are still a threat to residents in Shanghai, and the number of these outpatients tended to increase over the study period.

In our study, we found that although they accounted for relatively small proportions of the total outpatient visits, the numbers of visits for urogenital, blood, and endocrine diseases; mental and behavioural disorders; and neoplasms tended to increase from 2014 to 2018. The relatively low proportions of outpatients with these conditions may not be due to the actual incidence of these diseases but rather to the poor service in the community healthcare centres. For instance, the number of outpatients with neoplasms in this study was small, possibly because patients with these diseases directly visit higher-level hospitals. Interestingly, in our study, we found that symptoms affecting health but without diseases or injuries was a relatively common disease, especially in the groups aged 0–18 years (rank 1) and 19–39 years (rank 2). This indicated that general practitioners cannot diagnose many patients who seek medical services. Meanwhile, problems associated with medical equipment and other healthcare deficiencies were more common in suburban areas. Together, these findings suggest that disease diagnosis and treatment should be improved in the younger and suburban populations.

Unlike in all developed countries, which utilize primary care as the gatekeeper for the healthcare system, it has only been in recent years that China’s community healthcare institutions have started to receive attention and to improve in their ability to meet patient needs. However, because the referral system is not strict under the loosely regulated insurance system, more patients are inclined to initially visit higher-level hospitals. Community health care institutions are usually defined as playing a main role in disease prevention and control [32, 33]. Much work still needs to be done regarding the improvement of the medical functionality of community health care centres and the prevention and treatment of endemic diseases, which represents a gap between China and high-income countries [34, 35]. Based on the disease spectrum among outpatients in all community healthcare centres in Shanghai, we suggest that the delivery of primary care should be evidence-based and targeted at the services most commonly utilized by vulnerable groups.

## Limitations

As this is the first analysis of the data pertaining to community healthcare institutions, we intend to convene an expert panel to discuss ways in which to improve the primary care system in China. However, there are several limitations to this study. First, due to the nature of the outpatient records system, we could not access the comorbidities of the outpatients, which may lower the precision of the outpatient disease spectrum. Second, due to the statistical power, there may be disease spectrum disparities between this study and other domestic and international studies. Further published results from the outpatient data are expected. Third, because the population was stable over the study period, and we analysed the proportion instead of incidence or prevalence, both discounting and age-weighting were omitted from this study.

## Conclusions

Accurate assessment of the patterns of disease in community healthcare institutions is critical for making evidence-based decisions regarding primary healthcare development in China. The ranks of various diseases differed according to sex, age and regional characteristics, signifying that resources should be allocated based on these characteristics. To meet the patients’ needs and to develop the primary care system, the Shanghai government should focus on diseases with regionally high proportions, and targeted prevention and treatment efforts should be directed towards the most commonly encountered conditions.

## Data Availability

The data were outpatient data collected anonymously from the Shanghai information Center of the Health and Family Planning Commission. The access to data needs their consent.

## Abbreviations

NCDs: Non-communicable diseases
EHR: Electronic health record
YLDs: Years lived with disabilities
PM: Particulate matter
